# No causal association between COVID-19 and sepsis: a bidirectional two-sample Mendelian randomization study

**DOI:** 10.3389/fimmu.2023.1183489

**Published:** 2023-10-09

**Authors:** Hao Lu, Yu Cao, Ming Zhong

**Affiliations:** ^1^School of Medicine, Xiamen University, Xiamen, China; ^2^Department of Oral Histopathology, School and Hospital of Stomatology, China Medical University, Liaoning Province Key Laboratory of Oral Disease, Shenyang, Liaoning, China

**Keywords:** COVID-19, sepsis, Mendelian randomization, immune response, genetics

## Abstract

**Background:**

Sepsis and COVID-19 have a well-established observable relationship. Whether COVID-19 increases the likelihood of developing sepsis and whether patients with sepsis are at increased risk for COVID-19 infection is unknown. Using a bidirectional 2-sample Mendelian randomization (TSMR) analysis techniques in sizable cohorts, we sought to answer this question.

**Methods:**

The current study performed Mendelian randomization (MR) on publicly accessible genome-wide association study (GWAS) summary data in order to investigate the causal linkages between COVID-19 and sepsis. A Two-Sample MR(TSMR) analyses was performed. As instrumental variables, a COVID-19 dataset of single nucleotide polymorphisms (SNPs) with significance value smaller than 5*10^-8^ was employed and Sepsis dataset of SNPs with significance value smaller than 5*10^-7^was employed.

**Results:**

The results suggested that Very severe respiratory confirmed COVID-19(VSRC), hospitalized COVID-19(HC) and Infected COVID-19(IC) had no causal influence on sepsis risk using the inverse variance weighted (IVW) technique (VSRC OR = 1.000, 95% CI, 0.956-1.046, P = 0.996, HC OR = 0.976, 95% CI, 0.920-1.036, P = 0.430, IC OR = 0.923, 95% CI, 0.796-1.071, P = 0.291) and there was no causal effect of sepsis on the risk of VSRC, HC and IC (VSRC OR = 0.955, 95% CI, 0.844-1.173, P = 0.953, HC OR = 0.993, 95% CI, 0.859-1.147, P = 0.921, IC OR = 1.001, 95% CI, 0.959-1.045, P = 0.961).

**Conclusions:**

Our findings do not support a causal relationship between COVID-19 and sepsis risk, nor do they suggest a causal link between sepsis and COVID-19. The bidirectional relationship between COVID-19 and sepsis warrants further investigation in large cohorts.

## Introduction

The coronavirus disease 2019 (COVID19), also called as the severe acute respiratory syndrome coronavirus 2 (SARS-CoV2)-induced respiratory distress syndrome, was discovered in humans in late 2019 (1). SARS-CoV-2 infection is very heterogeneous in both susceptibility and severity, with clinical severity (2, 3) ranging from asymptomatic infection to fatal respiratory failure (4). Severe COVID-19 has been linked in several studies to older age, male sex, and a number of comorbidities, including obesity, type 2 diabetes, cardiovascular disease, and hypertension (5–12). Millions of people have been impacted by the ongoing COVID-19 epidemic, which poses a major risk to public health.

Sepsis is one of the main causes of infection-related death and is a severe, potentially fatal clinical syndrome. Bacterial pathogens are frequently implicated in the development of sepsis, but other pathogens such as viruses, fungi, and bacteria can also cause it (13). In the 2016 revision of the Surviving Sepsis Campaign(SSC), sepsis is now described as a potentially fatal organ failure brought on by a dysregulated host reaction to infection (14). According to information acquired from multiple cohorts, respiratory failure and the start of sepsis are the leading causes of mortality from COVID-19 (15). Sepsis has really been detected in nearly all patients who passed away in several of the groups that have been studied (16–18). While the sepsis statistics were inconsistently correlated with the bacterial discoveries in the microbiological work-up, recent research proposed SARS-CoV-2 as the causative agent of this systemic disease (19). Although the above study shows that there is an association between SARS-CoV-2 and sepsis, whether SARS-CoV-2 has a causal relationship with the occurrence and development of sepsis remains to be further verified.

The purpose of this study is to investigate the bidirectional causal effects between SARS-CoV-2 infection and sepsis development. Confounding factors are lessened by a method known as Mendelian randomization (MR) (20), which incorporates summary data from genome-wide association studies (GWAS). MR is a well-liked method for figuring out whether exposure and complicated outcomes have any causal relationships. The selection of instrumental variables (IVs) comprises genetic variations that are closely connected to exposure in order to infer causality (21). If the exposure is causal, the instrumental variables influencing the exposure will have an impact on the results. Elucidating the causal nature of this relationship is critical to understanding COVID-19 pathogenesis and improving treatment strategies.

## Materials and methods

### The MR research’s design

**Figure 1:**
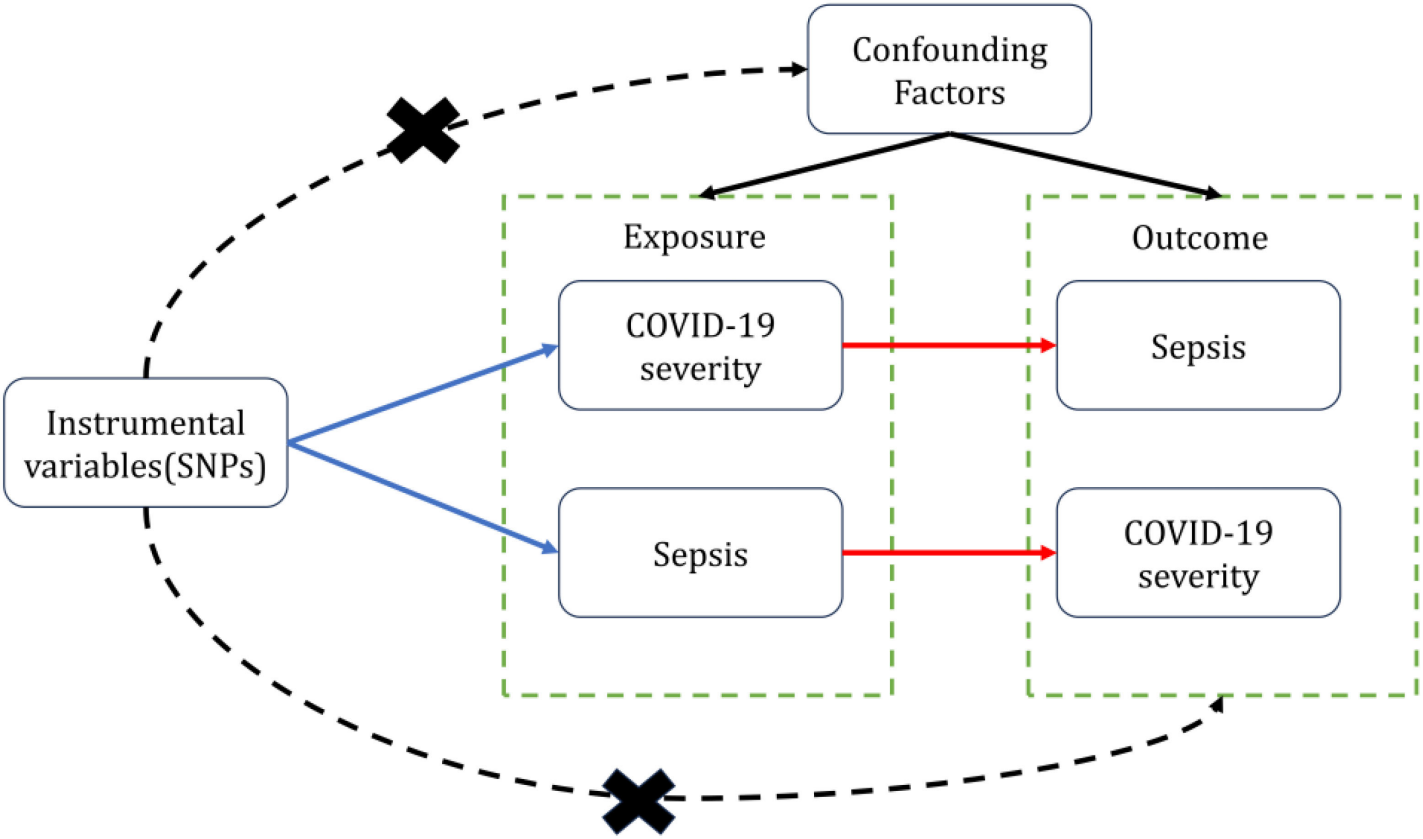
Two-sample bidirectional Mendelian randomization framework and assumptions of sepsis and COVID-19 severity.

We use COVID-19 severity including Very severe respiratory confirmed COVID-19(VSRC), hospitalized COVID-19(HC) and Infected COVID-19(IC) as “exposure,” respectively, and Sepsis as “outcome,” to screen out the instrumental variables for bidirectional Mendelian randomization analysis, To minimize type I error and assess heterogeneity, we adopted Bonferroni correction for significance thresholds and performed Cochran’s Q test. To validate the robustness of causal conclusions, we conducted sensitivity analyses including horizontal pleiotropy analysis using MR-Egger regression and leave-one-out analysis. We then performed reverse MR with Sepsis as “exposure” and COVID-19 severity as “outcomes.” Valid MR analysis requires the following key assumptions: (1) The genetic variants used as instrumental variables must demonstrate robust associations with the exposure; (2) The genetic instruments should not be correlated with any confounders of the exposure-outcome relationship; (3) The genetic variants shall affect the outcome only through the exposure, without exerting horizontal pleiotropic effects (**Figure 1**). In this study, bidirectional Mendelian randomization was used to evaluate the causal connection between COVID-19 severity and sepsis.

### Sources of data

The IEU Open GWAS with summary-level data, which includes 10,154 explicit sepsis cases and 454,764 controls, was used to obtain data on the genetic predisposition to sepsis. They were developed by the MRC Integrative Epidemiology Unit (MRC-IEU) consortium, which is based on the UK Biobank (http://www.nealelab.is/uk-biobank). We used information for COVID-19 from the COVID-19 Host Genetics Initiative (HGI, https://www.covid19hg.org/) Release 7, a worldwide partnership to support COVID-19 genetics research (22). To reduce the possibility of sample overlap with GWAS of sepsis, we chose the sets of GWAS summary statistics that did not include the UK Biobank (UKBB) sample. Three separate COVID-19 results were assessed: Hospitalized COVID-19 patients (n = 40929) compared with community-based controls (n = 1924400); Very severe respiratory confirmed COVID-19 patients (n = 17472) compared with population-based controls (n = 2357647); Infected COVID-19 population (n = 143839) compared with population based controls (n =2357647).

In order to ensure the accuracy and validity of the findings on the causal relationship between COVID-19 and sepsis, quality check techniques were used to choose the suitable instrument variables. A group of COVID-19 SNPs with P-values less than 5*10^-8^ and sepsis SNPs less than 5*10^-7^ were chosen as instrumental variables. Second, since considerable LD might result in skewed results, the lack of linkage disequilibrium (LD) among the chosen instrumental variable is a fundamental guiding principle of the MR technique. In the current study, the degree of LD between the additional SNPs was examined using the clumping approach [R2 0.01 and clump window = 10,000kb, with European reference panel from the 1000 Genomes Project (23)]. Third, a crucial component of MR is determining whether the effects of SNPs in exposure correspond to the same genotype as the effects on outcome. According to the notion, the instrumental variables wouldn’t contain palindromic SNPs. Fourth, in cases where GWAS data lacked SNPs associated with exposure, proxy SNPs with (R2 > 0.8) significantly associated with the relevant variations were chosen.

### The MR assumption

To reduce the effects of bias on the results, the MR technique must adhere to three key presumptions. First off, instrumental variables that alter exposure and result have no effect on other instrumental variables. Second, the variations of interest should be closely related to the study’s exposure. When evaluating how strongly instrumental variables and exposure relate to one another, the F value is frequently used. F = R^2^(n-k-1)/k(1-R^2^) is the formula for the F statistic (24). The sample size is N, the quantity of the instrumental variable is k, and the treatment variation, which the selected SNPs represent, is R2. When F value is less than 10, the relationship between the instrumental variable and the exposure is poor. Third, because instrumental variables only influence outcomes through exposure, there is no horizontal pleiotropy effect between instrumental variables and outcomes.

### MR analysis

To evaluate the causal effects of exposures on outcomes, we used a number of complimentary methods in this work, including the inverse variance weighted (IVW), the MR-Egger regression, the weighted median, the simple mode, and the weighted mode methods. The primary analytical method was the IVW approach.

When all chosen SNPs were valid IVs, the IVW technique would produce the most accurate findings for basic causal estimations. The Wald ratio estimations are weighted averaged using the IVW technique (25). Using the premise of Instrument Strength Independent of Direct Effect, the MR-Egger regression runs a weighted linear regression and generates a consistent causal estimate even if all of the genetic IVs are incorrect (InSIDE) (26). It has a poor level of accuracy, though, and is vulnerable to genetic variants. As the weighted median regression technique does not require the InSIDE hypothesis, it is immune to horizontal pleiotropic bias and provides a weighted median of the Wald ratio estimates (27). It has been established that the Weighted Median technique outperforms the MR-Egger regression in various ways, offering reduced type I error and more causal estimate power. By grouping the SNPs into subsets based on similarities in their causal effects, the causal effect of the subgroup with the most SNPs is calculated using the Weighted Mode approach (28). The simple mode approach is also less biased than other methods while being less precise since it can reduce bias (28).

To assess the potential for horizontal pleiotropy, we used the MR-Egger regression. The intercept term of MR-Egger regression represents the average pleiotropic impact of the IVs (26). To determine whether pleiotropy existed, the MR Pleiotropy REsidual Sum and Outlier (MR-PRESSO) test was also performed (29). Its tasks include identifying horizontal pleiotropy, eliminating outliers to account for horizontal pleiotropy, and determining whether there are any discernible variations in the causal effects between the periods before and after outlier removal. We used the MR-Egger regression and the IVW technique to identify heterogeneity, and Cochran’s Q was used to measure the heterogeneities. To prevent horizontal pleiotropy brought on by a single SNP, a leave-one-out analysis was carried out, which systematically drops one SNP at a time.

Multiple comparisons were taken into consideration using the Bonferroni method, and a p-value of less than 0.05 demonstrated significant support for causal links. With three sets of SNPs analyzed, this resulted in a adjusted significance threshold of 0.05/3 = 0.0167. Only p-values below this Bonferroni-corrected cut-off of 0.0167 were considered statistically significant. The packages “TwoSampleMR” (30) and “MRPRESSO” in R version 4.2.1 were used for every analysis.

## Results

### Causal effects of COVID-19 on sepsis

**Figure 2:**
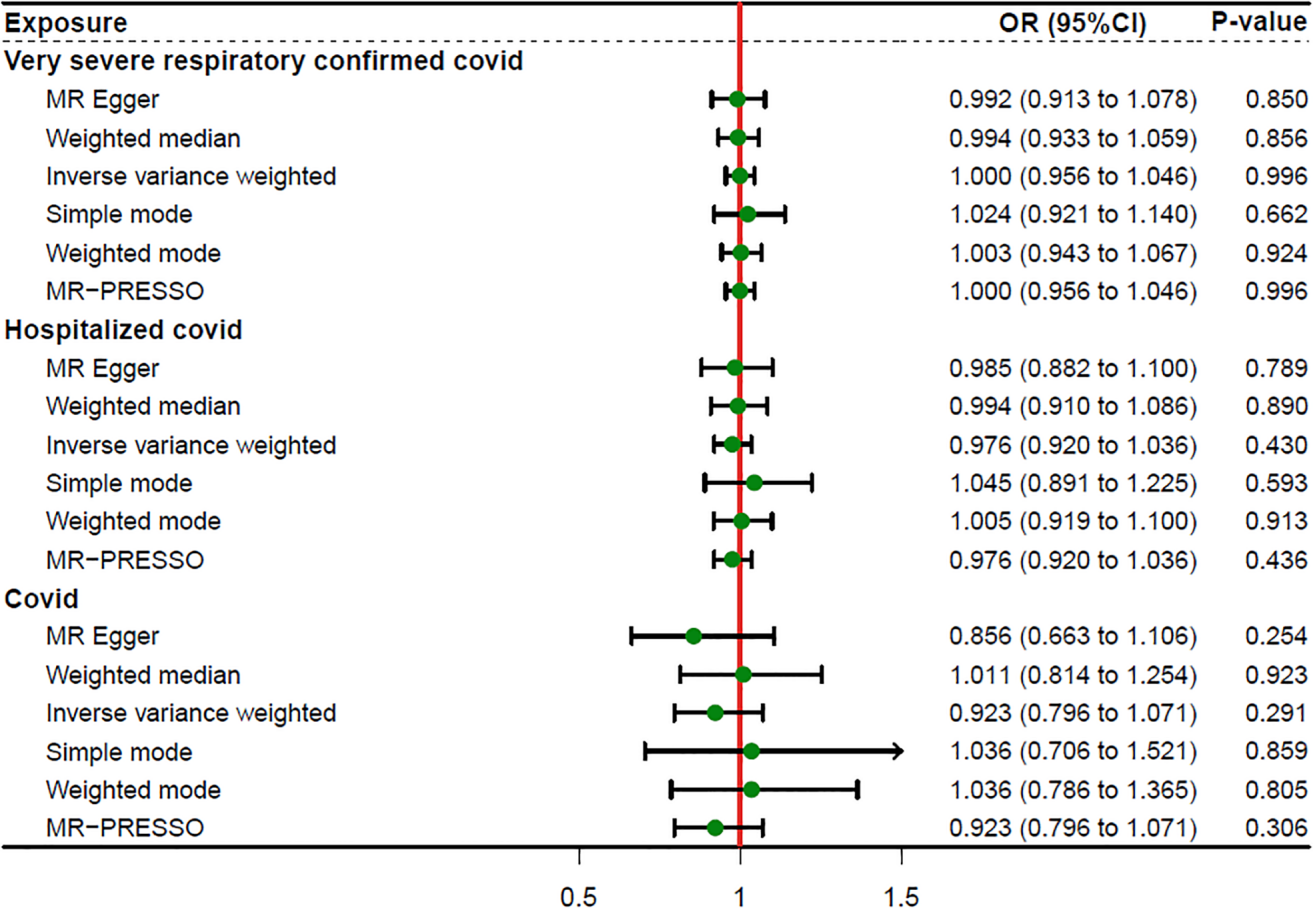
Forest plot showing MR results of the effect of COVID-19 on sepsis.

We incorporated 30, 36, and 17 independent SNPs with significant p-value less than 5×10^-8^ as IV SNPs for VSRC, HC and IC and **Supplementary Table S1** contains extensive information on the instrumental variables. Our TSMR analysis revealed that sepsis and COVID-19 do not appear to be significantly causally related. When setting COVID traits as the exposure, COVID-19 was no causally associated with sepsis, as shown in **Figure 2**. The results of IVW analyses demonstrated that VSRC (odds ratio (OR) = 1.000, 95% confidence interval (CI),0.956-1.046, P = 0.996) HC (OR = 0.976, 95% CI, 0.920-1.036, P = 0.430) and IC (OR = 0.923, 95% CI, 0.796-1.071, P = 0.291) were no significantly associated with the risk of sepsis. The MR-Egger, Weighted Median, Simple Mode, MR-PRESSO, and Weighted Mode approaches also produced reliable findings. There was no heterogeneity between the individual SNPs, according to the heterogeneity test. The results of the MR-Egger regression and MR-PRESSO global test indicated that it was unlikely that horizontal pleiotropy would skew the cause of sepsis in COVID-19 (**Table 1**). Leave-one-out analysis revealed that no one SNP was responsible for the causative estimates of COVID-19 and sepsis. In **Supplementary Figure S1**, the leave-one-out analysis plots were displayed.

**Table 1:** Heterogeneity test and pleiotropy test of genetic variants when COVID-19 as exposure.

Exposure	VSRC	HC	IC
Pleiotropy test P-value	0.819	0.856	0.488
MR Egger heterogeneity test P-value	0.170	0.201	0.388
IVW heterogeneity test P-value	0.203	0.235	0.422
MR-PRESSO global test P-value	0.237	0.252	0.373

### Causal effects of sepsis on COVID-19

**Figure 3:**
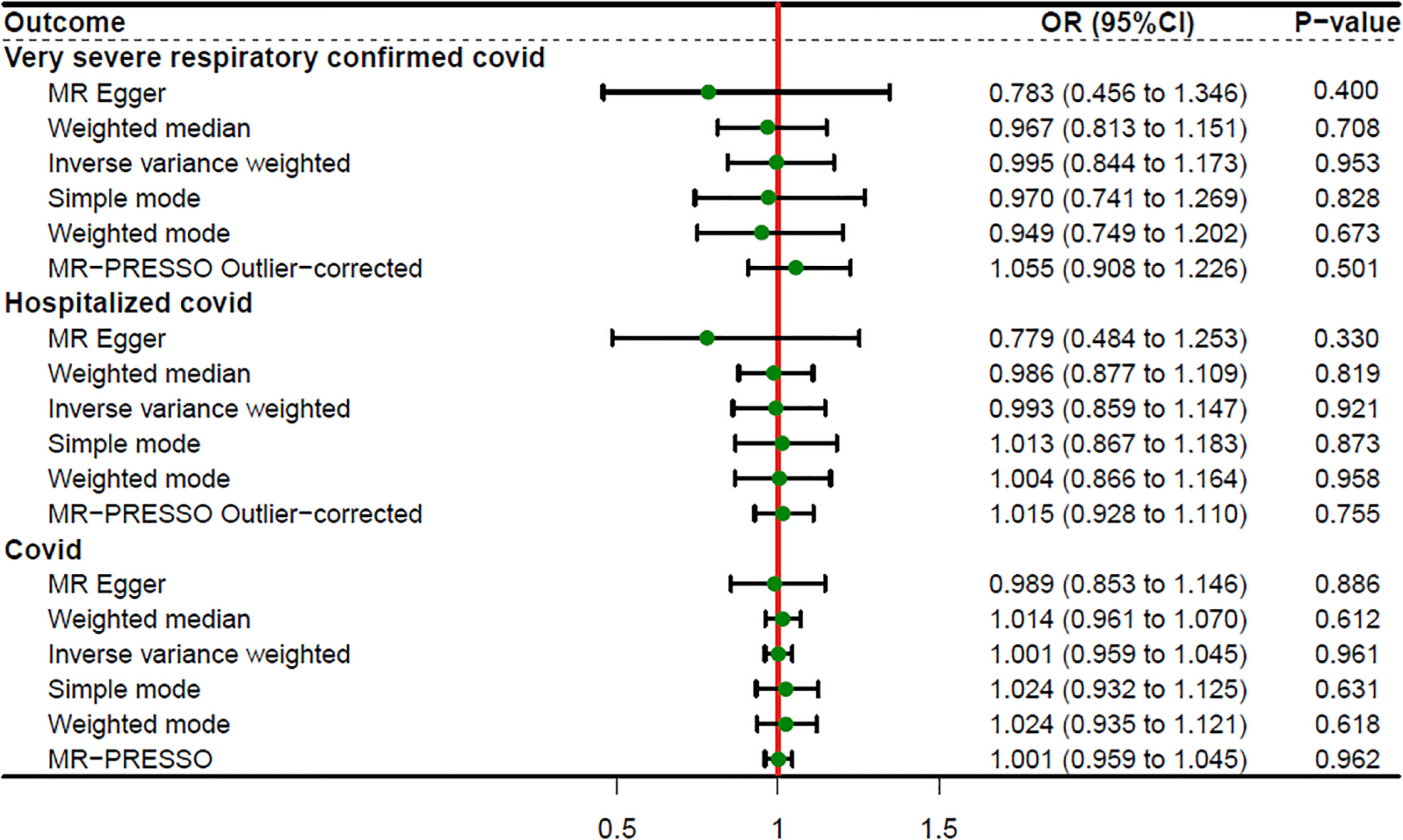
Forest plot showing MR results of the effect of sepsis on COVID-19.

When setting COVID traits as the outcome, 11 independent SNPs with significant p-value less than 5×10^-7^ were extracted as IV. Detailed information on these IVs is provided in **Supplementary Table S2**. Sepsis was not causally associated with COVID-19, as shown in **Figure 3**. There was no evidence of a connection between sepsis and the risk of COVID-19 based on the IVW approach (VSRC: OR=0.995, 95% CI: 0.844-1.173, P = 0.953; HC: OR=0.993, 95% CI: 0.859-1.147, P = 0.921; IC: OR=1.001, 95% CI: 0.959-1.045, P = 0.961). The MR-Egger, Weighted Median, Simple Mode, MR-PRESSO, and Weighted Mode approaches also produced consistent findings (**Table 2** and **Supplementary Figure S2**).

**Table 2:** Heterogeneity test and pleiotropy test of genetic variants when sepsis as exposure.

Outcome	VSRC	HC	IC
Pleiotropy test P-value	0.386	<0.00333	0.270
MR Egger heterogeneity test P-value	0.0472	0.00109	0.212
IVW heterogeneity test P-value	0.0446	0.000579	0.281
MR-PRESSO global test P-value	0.0467	0.252	0.373

## Discussion

This was the first research to use a variety of complementary MR techniques to examine the bidirectional causal relationship between sepsis and COVID-19. A genetically anticipated COVID-19 association with sepsis in people of European ancestry was not seen in our two-sample MR investigation. The reverse MR study also showed no proof that COVID-19 and genetic susceptibility to sepsis were connected.

There have been earlier epidemiological studies that found a connection between sepsis and COVID-19. According to a recent meta-analysis, a sizable majority of hospitalized patients have COVID-19-related sepsis based on Sepsis-3; 77.9% of adult patients in the ICU had viral sepsis (31). A prospectively recruited, multicentric study’s retrospective analysis also revealed that during the COVID-19 pandemic, the raw 30-day death rate for sepsis patients rose (32). Nevertheless, this cohort study has drawbacks, such as a limited patient cohort and recruitment bias, which might prevent the detection of important effects. As a result, drawing conclusions about the causes of COVID-19 and sepsis merely from past research is challenging.

Several reports have shown since the beginning of the pandemic that while COVID-19 has certain distinctive characteristics, many of its acute signs are comparable to sepsis brought on by other viruses (19, 33–35). The correlation between COVID-19 and sepsis in observational studies can be explained by a number of factors. As a result of a number of potential processes, including immunological dysregulation, respiratory failure that results in hypoxemia, and metabolic acidosis brought on by circulatory malfunction, research has shown that the virus itself probably produces a sepsis syndrome (36). The most likely explanation is that sepsis-like cytokine storms are linked to COVID-19 illness (33, 37). In sepsis, there are two phases that occur one after the other. The first phase is a hyper-inflammatory phase, and the second is an immunosuppressive phase (38). Several indicators, including C-reactive protein (CRP), procalcitonin, tumor necrosis factors (TNF), interleukin (IL) 1 and IL-6, are present throughout the hyperinflammatory phase. Pro-inflammatory cytokines TNF, IL-1, and IL-6 are generated as part of the body’s early response to injury or illness (39). The liver produces CRP in response to infection and is induced to do so by IL-6. The body produces procalcitonin, which is regarded as the most effective sign of severe systemic inflammation. A number of these indicators are recognized as sepsis biomarkers and can be utilized to help diagnose and treat sepsis patients (40). Similar to this, it has been shown that these markers are elevated in patients with severe COVID-19 (19, 41–43). This highlights that the exact mechanisms leading to hyperinflammation and organ damage in COVID-19 remain incompletely understood. More research is still needed to elucidate the complex immunopathology of severe COVID-19 illness.

Our study conclusions indicate clinicians should be cautious about relying solely on interventions targeting sepsis to improve COVID-19 outcomes. While managing secondary sepsis is still important, our findings underscore the need to better understand the distinct mechanisms leading to critical COVID-19 pneumonia apart from sepsis. Ultimately, our results point to the need for further investigation into the biological pathways underlying progression to acute respiratory distress syndrome and multiple organ failure in COVID-19.

The inference of causation between COVID-19 and sepsis in both directions was ensured by the bidirectional analysis, which is a strength of the current bidirectional MR study. Compared to previous MR studies that largely examined COVID-19 hospitalization or mortality, our study outcome of severe vs. mild COVID-19 phenotypes allows us to detect more nuanced effects on disease progression. We also conducted thorough sensitivity analyses to assess pleiotropy that were not performed uniformly across prior works. There are a few restrictions with this study, though. The research group with European ancestry was included in the MR analysis, to start. As a result, it is still unknown whether the findings can be considered representative of the entire population. Second, although it is difficult to assess the degree of sample overlap, individuals in the exposure and outcome research likely overlapped. Luckily, the powerful instruments used in this study (F statistic significantly higher than 10) should reduce any potential bias resulting from sample overlap (44). Third, Pleiotropic effects of genetic variants cannot be fully ruled out, although sensitivity analyses were conducted to assess this. Fourth, variants associated with VSRC showed some evidence of bias based on MR Egger and IVW heterogeneity test P-values <0.05. This suggests pleiotropy and linkage disequilibrium may impact these VSRC results. Though we attempted to minimize confounding through outlier removal and robust methods, some residual bias cannot be excluded. Further analyses in larger datasets and with additional quality control steps could provide more definitive results. Fifth, previous observational studies have suggested an association between sepsis and COVID-19 (45), whereas our Mendelian randomization analysis found no evidence for a causal relationship; synthesizing these complementary lines of evidence across the broader literature highlights the need for multiple approaches when assessing causality, and our MR findings alone cannot definitively rule out a causal effect. Finally, the sepsis GWAS may capture susceptibility to infections beyond just bacterial sepsis.

## Conclusions

In conclusion, our findings do not support a causal relationship between COVID-19 and sepsis risk, nor do they suggest a causal link between sepsis and COVID-19. Verification of the study’s findings will require updated MR analysis based on more genetic instruments and larger scale GWAS summary data.

## Data availability statement

Publicly available datasets were analyzed in this study. This data can be found here: UK Biobank (http://www.nealelab.is/uk-biobank). COVID-19 Host Genetics Initiative Release 7 (HGI, https://www.covid19hg.org/).

## Author contributions

YC and MZ conceived the presented idea. HL and YC performed the manuscript writing. YC and MZ was involved in acquisition and processing of data. HL was involved in interpretation of data. YC and HL have contributed equally to this work and share first authorship. All authors contributed to the article and approved the submitted version.
