# Supplementary material for: No causal association between COVID-19 and sepsis: a bidirectional two-sample Mendelian randomization study
Source: Front Immunol. 2023 Oct 9;14:1183489. doi: 10.3389/fimmu.2023.1183489 (PMC10591217; doi:10.3389/fimmu.2023.1183489)
Supplement: Supplementary file 1 [file DataSheet_1.docx]

**Supplementary Table S1 Included SNPs in MR analyses for each COVID-19 trait on sepsis**

| SNP | chr | pos | effect_allele | other_allele | eaf | beta.exposure | beta.outcome | se.exposure | se.outcome | pval.exposure | pval.outcome | Fval | exposure |
| --- | --- | --- | --- | --- | --- | --- | --- | --- | --- | --- | --- | --- | --- |
| rs10066378 | 5 | 1.32E+08 | C | T | 0.1183 | 0.11874 | 0.001686 | 0.018594 | 0.021892 | 1.70E-10 | 0.88 | 2192.277 | Very severe respiratory confirmed covid |
| rs10850097 | 12 | 1.13E+08 | T | C | 0.691 | 0.095523 | 0.001216 | 0.013989 | 0.014821 | 8.59E-12 | 0.77 | 2907.13 | Very severe respiratory confirmed covid |
| rs1120591 | 8 | 61537523 | C | T | 0.4175 | 0.076004 | -0.0075 | 0.01334 | 0.014702 | 1.22E-08 | 0.51 | 2093.937 | Very severe respiratory confirmed covid |
| rs1123573 | 2 | 60707588 | G | A | 0.3426 | -0.12036 | -0.01282 | 0.014376 | 0.014637 | 5.64E-17 | 0.4 | 4881.363 | Very severe respiratory confirmed covid |
| rs1128175 | 6 | 31150435 | G | A | 0.7383 | -0.11827 | 0.000586 | 0.01601 | 0.01705 | 1.50E-13 | 0.8 | 4038.834 | Very severe respiratory confirmed covid |
| rs11614702 | 12 | 1.33E+08 | A | G | 0.4805 | 0.099671 | 0.012492 | 0.013058 | 0.014173 | 2.29E-14 | 0.39 | 3704.182 | Very severe respiratory confirmed covid |
| rs116415481 | 1 | 1.55E+08 | T | C | 0.02953 | 0.2352 | 0.04404 | 0.039432 | 0.042452 | 2.45E-09 | 0.46 | 2363.828 | Very severe respiratory confirmed covid |
| rs117169628 | 16 | 89262657 | A | G | 0.1238 | 0.14967 | -0.02235 | 0.019651 | 0.020211 | 2.60E-14 | 0.24 | 3629.323 | Very severe respiratory confirmed covid |
| rs12585036 | 13 | 1.14E+08 | T | C | 0.2165 | 0.14326 | 0.001535 | 0.016086 | 0.017559 | 5.28E-19 | 0.98 | 5210.719 | Very severe respiratory confirmed covid |
| rs12610495 | 19 | 4717672 | G | A | 0.3132 | 0.24613 | 0.004728 | 0.015302 | 0.015518 | 3.28E-58 | 0.58 | 19886.87 | Very severe respiratory confirmed covid |
| rs12614007 | 2 | 57316503 | A | G | 0.7529 | 0.088893 | 0.013472 | 0.01604 | 0.0166 | 2.99E-08 | 0.48 | 2191.494 | Very severe respiratory confirmed covid |
| rs17279437 | 3 | 45814094 | A | G | 0.08753 | -0.17222 | 1.67E-06 | 0.025182 | 0.02299 | 7.98E-12 | 0.81 | 3537.705 | Very severe respiratory confirmed covid |
| rs17763742 | 3 | 45846769 | G | A | 0.09222 | 0.72793 | -0.01048 | 0.023628 | 0.02792 | 1.00E-200 | 0.58 | 72351.73 | Very severe respiratory confirmed covid |
| rs2070788 | 21 | 42841988 | A | G | 0.5819 | -0.07615 | 0.007077 | 0.013356 | 0.014372 | 1.19E-08 | 0.780001 | 2102.923 | Very severe respiratory confirmed covid |
| rs2496644 | 6 | 41482745 | C | A | 0.9185 | -0.34017 | -0.01281 | 0.033282 | 0.059065 | 1.60E-24 | 0.9 | 13101.92 | Very severe respiratory confirmed covid |
| rs28368148 | 9 | 21206605 | G | C | 0.03451 | 0.44713 | 0.132339 | 0.065915 | 0.072872 | 1.17E-11 | 0.11 | 10034.64 | Very severe respiratory confirmed covid |
| rs2897075 | 7 | 99630342 | T | C | 0.3804 | 0.077143 | 0.040924 | 0.013338 | 0.014714 | 7.30E-09 | 0.0029 | 2090.65 | Very severe respiratory confirmed covid |
| rs2924480 | 11 | 34529831 | C | T | 0.3418 | -0.13204 | -0.01184 | 0.014454 | 0.014965 | 6.52E-20 | 0.43 | 5875.944 | Very severe respiratory confirmed covid |
| rs2983793 | 10 | 81445802 | G | A | 0.4299 | 0.086404 | 0.012634 | 0.014206 | 0.014956 | 1.18E-09 | 0.68 | 2729.573 | Very severe respiratory confirmed covid |
| rs343320 | 3 | 1.46E+08 | A | G | 0.05542 | 0.15312 | 0.010857 | 0.027083 | 0.026073 | 1.57E-08 | 0.87 | 1828.744 | Very severe respiratory confirmed covid |
| rs35617599 | 19 | 50874794 | A | G | 0.3144 | 0.095938 | 0.000882 | 0.01398 | 0.015133 | 6.77E-12 | 0.95 | 2960.587 | Very severe respiratory confirmed covid |
| rs35705950 | 11 | 1241221 | T | G | 0.1003 | -0.16173 | -0.00356 | 0.022234 | 0.022486 | 3.49E-13 | 0.9 | 3524.936 | Very severe respiratory confirmed covid |
| rs3848456 | 17 | 47940623 | A | C | 0.04567 | 0.29209 | 0.05204 | 0.033237 | 0.043072 | 1.52E-18 | 0.19 | 5568.282 | Very severe respiratory confirmed covid |
| rs41264915 | 1 | 1.55E+08 | G | A | 0.08094 | -0.20336 | -0.01886 | 0.021406 | 0.022513 | 2.09E-21 | 0.37 | 4600.816 | Very severe respiratory confirmed covid |
| rs41264931 | 1 | 1.55E+08 | C | G | 0.01419 | -0.29613 | 0.023295 | 0.049397 | 0.04951 | 2.04E-09 | 0.59 | 1827.779 | Very severe respiratory confirmed covid |
| rs550057 | 9 | 1.36E+08 | T | C | 0.2763 | 0.11508 | -0.04676 | 0.015006 | 0.016266 | 1.73E-14 | 8.50E-05 | 3956.962 | Very severe respiratory confirmed covid |
| rs646327 | 19 | 49209851 | G | A | 0.4211 | -0.09538 | 0.018633 | 0.013376 | 0.014214 | 1.00E-12 | 0.019 | 3310.724 | Very severe respiratory confirmed covid |
| rs74324246 | 21 | 35344479 | A | G | 0.09848 | 0.18866 | -0.02133 | 0.022387 | 0.026272 | 3.54E-17 | 0.27 | 4726.644 | Very severe respiratory confirmed covid |
| rs7528403 | 1 | 65382792 | G | T | 0.7404 | -0.0974 | 0.009185 | 0.016858 | 0.018338 | 7.58E-09 | 0.66 | 2719.921 | Very severe respiratory confirmed covid |
| rs9636867 | 21 | 34609944 | G | A | 0.3582 | 0.18972 | -0.03033 | 0.013868 | 0.015356 | 1.32E-42 | 0.04 | 12505.91 | Very severe respiratory confirmed covid |
| rs10066378 | 5 | 1.32E+08 | C | T | 0.1352 | 0.07838 | 0.001686 | 0.012291 | 0.021892 | 1.81E-10 | 0.88 | 2827.434 | Hospitalized covid |
| rs10774679 | 12 | 1.13E+08 | T | C | 0.6588 | 0.083578 | -0.0037 | 0.009497 | 0.014714 | 1.36E-18 | 0.51 | 6191.242 | Hospitalized covid |
| rs11208552 | 1 | 65412830 | T | G | 0.6248 | -0.05736 | -0.00152 | 0.009892 | 0.01556 | 6.70E-09 | 0.88 | 3036.074 | Hospitalized covid |
| rs1123573 | 2 | 60707588 | G | A | 0.3595 | -0.08188 | -0.01282 | 0.010155 | 0.014637 | 7.45E-16 | 0.4 | 6086.7 | Hospitalized covid |
| rs117169628 | 16 | 89262657 | A | G | 0.1308 | 0.10477 | -0.02235 | 0.013914 | 0.020211 | 5.09E-14 | 0.24 | 4917.586 | Hospitalized covid |
| rs11790730 | 9 | 33425785 | C | T | 0.1886 | 0.07587 | 0.009892 | 0.012179 | 0.017457 | 4.68E-10 | 0.54 | 3468.55 | Hospitalized covid |
| rs12151726 | 2 | 1.98E+08 | T | C | 0.396 | 0.057323 | -0.02069 | 0.009749 | 0.014297 | 4.11E-09 | 0.085 | 3094.129 | Hospitalized covid |
| rs12585036 | 13 | 1.14E+08 | T | C | 0.2081 | 0.10643 | 0.001535 | 0.011083 | 0.017559 | 7.75E-22 | 0.98 | 7364.787 | Hospitalized covid |
| rs12602210 | 17 | 65813731 | A | G | 0.1007 | 0.08381 | 0.016848 | 0.015238 | 0.025471 | 3.79E-08 | 0.43 | 2503.478 | Hospitalized covid |
| rs12610495 | 19 | 4717672 | G | A | 0.2883 | 0.16432 | 0.004728 | 0.010752 | 0.015518 | 9.89E-53 | 0.58 | 22020.48 | Hospitalized covid |
| rs12660421 | 6 | 41488378 | A | G | 0.04895 | 0.25373 | 0.003316 | 0.021365 | 0.059357 | 1.57E-32 | 0.97 | 11851.57 | Hospitalized covid |
| rs139589338 | 1 | 1.55E+08 | G | A | 0.01878 | 0.20783 | -0.04843 | 0.036889 | 0.053478 | 1.76E-08 | 0.47 | 3133.543 | Hospitalized covid |
| rs149533170 | 9 | 21172825 | A | G | 0.00673 | 0.33758 | 0.118553 | 0.054755 | 0.071844 | 7.03E-10 | 0.16 | 2998.909 | Hospitalized covid |
| rs17197276 | 6 | 31146340 | T | C | 0.09522 | 0.10448 | -0.0419 | 0.01553 | 0.028524 | 1.72E-11 | 0.056999 | 3703.569 | Hospitalized covid |
| rs17279437 | 3 | 45814094 | A | G | 0.09472 | -0.11822 | 1.67E-06 | 0.017698 | 0.02299 | 2.39E-11 | 0.81 | 4721.869 | Hospitalized covid |
| rs17412601 | 3 | 1.01E+08 | C | T | 0.3387 | -0.06815 | 0.015519 | 0.009782 | 0.014862 | 3.24E-12 | 0.056 | 4097.224 | Hospitalized covid |
| rs17763742 | 3 | 45846769 | G | A | 0.07939 | 0.52547 | -0.01048 | 0.016794 | 0.02792 | 1.00E-200 | 0.58 | 82659.91 | Hospitalized covid |
| rs186910 | 3 | 1.46E+08 | G | A | 0.0728 | 0.099001 | 0.010301 | 0.016465 | 0.025849 | 1.82E-09 | 0.91 | 2603.9 | Hospitalized covid |
| rs2068205 | 6 | 33058583 | C | T | 0.6296 | 0.054297 | -0.02423 | 0.009649 | 0.014376 | 1.83E-08 | 0.034 | 2706.14 | Hospitalized covid |
| rs2897075 | 7 | 99630342 | T | C | 0.3651 | 0.052287 | 0.040924 | 0.009289 | 0.014714 | 1.82E-08 | 0.0029 | 2494.139 | Hospitalized covid |
| rs35705950 | 11 | 1241221 | T | G | 0.09653 | -0.10595 | -0.00356 | 0.015857 | 0.022486 | 2.37E-11 | 0.9 | 3855.622 | Hospitalized covid |
| rs383510 | 21 | 42858367 | C | T | 0.551 | -0.05397 | -0.00201 | 0.009455 | 0.014259 | 1.14E-08 | 0.780001 | 2836.26 | Hospitalized covid |
| rs3848456 | 17 | 47940623 | A | C | 0.04098 | 0.19792 | 0.05204 | 0.021321 | 0.043072 | 1.65E-20 | 0.19 | 6069.927 | Hospitalized covid |
| rs41264915 | 1 | 1.55E+08 | G | A | 0.1003 | -0.14357 | -0.01886 | 0.01445 | 0.022513 | 2.91E-23 | 0.37 | 7338.545 | Hospitalized covid |
| rs4403445 | 8 | 61432007 | A | G | 0.4007 | 0.058447 | -0.0077 | 0.009034 | 0.014702 | 9.82E-11 | 0.5 | 3229.732 | Hospitalized covid |
| rs5023077 | 12 | 1.33E+08 | C | T | 0.521 | -0.06962 | -0.01094 | 0.009108 | 0.014192 | 2.11E-14 | 0.46 | 4766.323 | Hospitalized covid |
| rs550057 | 9 | 1.36E+08 | T | C | 0.2474 | 0.098795 | -0.04676 | 0.010301 | 0.016266 | 8.74E-22 | 8.50E-05 | 7169.362 | Hospitalized covid |
| rs56163354 | 10 | 81724418 | C | T | 0.1154 | 0.080329 | 0.016117 | 0.012706 | 0.023734 | 2.58E-10 | 0.62 | 2592.598 | Hospitalized covid |
| rs638294 | 19 | 50863023 | G | A | 0.33 | 0.086512 | 0.000255 | 0.009454 | 0.015138 | 5.67E-20 | 0.91 | 6525.99 | Hospitalized covid |
| rs646327 | 19 | 49209851 | G | A | 0.4761 | -0.0689 | 0.018633 | 0.009331 | 0.014214 | 1.54E-13 | 0.019 | 4664.761 | Hospitalized covid |
| rs717624 | 7 | 22894487 | C | T | 0.6216 | -0.05379 | -0.00011 | 0.00947 | 0.014386 | 1.34E-08 | 0.85 | 2678.986 | Hospitalized covid |
| rs7515509 | 1 | 77949123 | A | G | 0.3627 | 0.058384 | 0.003591 | 0.009662 | 0.014516 | 1.52E-09 | 0.36 | 3101.911 | Hospitalized covid |
| rs76608815 | 21 | 35353264 | T | C | 0.09215 | 0.13931 | -0.01883 | 0.015055 | 0.026234 | 2.17E-20 | 0.33 | 6402.533 | Hospitalized covid |
| rs7671107 | 4 | 25449225 | A | G | 0.7204 | -0.07412 | -0.01405 | 0.010985 | 0.017467 | 1.51E-11 | 0.6 | 4359.106 | Hospitalized covid |
| rs7949972 | 11 | 34502042 | T | C | 0.3477 | -0.0931 | -0.00341 | 0.009188 | 0.014643 | 3.94E-24 | 0.81 | 7757.938 | Hospitalized covid |
| rs9636867 | 21 | 34609944 | G | A | 0.3447 | 0.14045 | -0.03033 | 0.009401 | 0.015356 | 1.83E-50 | 0.04 | 17671.68 | Hospitalized covid |
| rs10774675 | 12 | 1.13E+08 | T | C | 0.6952 | 0.033726 | 0.000483 | 0.00476 | 0.014803 | 1.38E-12 | 0.719999 | 1206.401 | Covid |
| rs1123573 | 2 | 60707588 | G | A | 0.3638 | -0.02794 | -0.01282 | 0.004646 | 0.014637 | 1.83E-09 | 0.4 | 903.9381 | Covid |
| rs12610495 | 19 | 4717672 | G | A | 0.289 | 0.059673 | 0.004728 | 0.005092 | 0.015518 | 1.02E-31 | 0.58 | 3665.956 | Covid |
| rs12972221 | 19 | 50879140 | T | G | 0.3109 | 0.02932 | 0.003356 | 0.004666 | 0.015144 | 3.30E-10 | 0.93 | 921.7624 | Covid |
| rs17860169 | 21 | 34613301 | G | A | 0.3422 | 0.041961 | -0.03039 | 0.00461 | 0.015341 | 8.90E-20 | 0.039 | 1984.44 | Covid |
| rs184781326 | 14 | 29459234 | G | A | 0.04067 | -0.07513 | 0.022546 | 0.012375 | 0.031647 | 1.27E-09 | 0.4 | 1102.124 | Covid |
| rs2260685 | 3 | 1.95E+08 | C | T | 0.4632 | 0.027161 | 0.020051 | 0.004548 | 0.014243 | 2.33E-09 | 0.26 | 918.0366 | Covid |
| rs2290859 | 3 | 1.02E+08 | T | C | 0.3402 | -0.05132 | 0.015985 | 0.004726 | 0.014873 | 1.79E-27 | 0.052 | 2961.044 | Covid |
| rs35044562 | 3 | 45909024 | G | A | 0.07836 | 0.14724 | -0.00195 | 0.008023 | 0.027966 | 3.23E-75 | 0.82 | 7857.737 | Covid |
| rs41435745 | 6 | 41490382 | C | G | 0.04977 | 0.088902 | 0.006823 | 0.01086 | 0.059318 | 2.70E-16 | 0.98 | 1871.424 | Covid |
| rs505922 | 9 | 1.36E+08 | C | T | 0.3539 | 0.085924 | -0.03976 | 0.004488 | 0.015217 | 1.05E-81 | 0.00043 | 8474.345 | Covid |
| rs721917 | 10 | 81706324 | G | A | 0.4298 | 0.028024 | 0.011498 | 0.004373 | 0.014374 | 1.48E-10 | 0.51 | 963.2725 | Covid |
| rs73062389 | 3 | 45835417 | A | G | 0.05014 | 0.20325 | 0.015064 | 0.010166 | 0.029841 | 6.41E-89 | 0.74 | 9882.012 | Covid |
| rs75586969 | 21 | 35265459 | T | C | 0.08383 | 0.043554 | -0.00851 | 0.007626 | 0.02754 | 1.12E-08 | 0.49 | 729.0986 | Covid |
| rs7949972 | 11 | 34502042 | T | C | 0.3511 | -0.02867 | -0.00341 | 0.0045 | 0.014643 | 1.87E-10 | 0.81 | 937.4447 | Covid |
| rs914615 | 1 | 1.55E+08 | G | A | 0.5515 | -0.0248 | -0.01256 | 0.00437 | 0.014201 | 1.38E-08 | 0.26 | 761.2662 | Covid |
| rs9916158 | 17 | 38182229 | T | G | 0.3634 | 0.025686 | -0.00979 | 0.004495 | 0.014688 | 1.10E-08 | 0.46 | 763.8449 | Covid |

**Supplementary Table S2 Included SNPs in MR analyses for sepsis on each COVID-19 trait**

| SNP | chr | pos | effect_allele | other_allele | eaf | beta.exposure | beta.outcome | se.exposure | se.outcome | pval.exposure | pval.outcome | Fvalue | outcome |
| --- | --- | --- | --- | --- | --- | --- | --- | --- | --- | --- | --- | --- | --- |
| rs10773497 | 12 | 128644754 | C | T | 0.478062 | 0.0728594 | -0.0085156 | 0.0142395 | 0.013501 | 4.00E-07 | 0.52822 | 1229.59 | Very severe respiratory confirmed covid |
| rs10790976 | 11 | 128778315 | T | C | 0.111758 | 0.118333 | 0.011581 | 0.0226516 | 0.019729 | 1.50E-07 | 0.55719 | 1290.52 | Very severe respiratory confirmed covid |
| rs117830939 | 8 | 88806713 | T | C | 0.047662 | 0.188806 | -0.024444 | 0.0337571 | 0.036004 | 8.50E-09 | 0.4972 | 1502.923 | Very severe respiratory confirmed covid |
| rs12203592 | 6 | 396321 | T | C | 0.219623 | 0.0470769 | 0.04663 | 0.0168804 | 0.030184 | 3.90E-09 | 0.12238 | 351.9347 | Very severe respiratory confirmed covid |
| rs12639198 | 3 | 187891885 | A | G | 0.284192 | 0.0758272 | 0.011485 | 0.0160451 | 0.016249 | 4.40E-07 | 0.47968 | 1085.45 | Very severe respiratory confirmed covid |
| rs3130320 | 6 | 32223258 | C | T | 0.623606 | -0.0771445 | -0.0026233 | 0.0145878 | 0.020757 | 8.30E-10 | 0.89943 | 1296.916 | Very severe respiratory confirmed covid |
| rs34613444 | 2 | 58499373 | G | T | 0.058915 | -0.149106 | 0.03182 | 0.0303123 | 0.026246 | 3.50E-07 | 0.22537 | 1144.066 | Very severe respiratory confirmed covid |
| rs722266 | 11 | 92034702 | C | T | 0.817568 | -0.100221 | 0.042222 | 0.0184403 | 0.017543 | 7.80E-08 | 0.016096 | 1391.168 | Very severe respiratory confirmed covid |
| rs748154 | 8 | 134708408 | A | G | 0.317744 | 0.0754683 | -0.0017953 | 0.015242 | 0.014398 | 3.00E-07 | 0.90077 | 1145.939 | Very severe respiratory confirmed covid |
| rs78081797 | 3 | 94777466 | T | C | 0.060878 | 0.14461 | 0.068788 | 0.0319317 | 0.034099 | 4.20E-07 | 0.043661 | 1109.565 | Very severe respiratory confirmed covid |
| rs9287883 | 2 | 168396255 | A | G | 0.311151 | -0.0711087 | -0.026412 | 0.0153409 | 0.014701 | 3.40E-07 | 0.07239 | 1005.581 | Very severe respiratory confirmed covid |
| rs10773497 | 12 | 128644754 | C | T | 0.478062 | 0.0728594 | 0.0032271 | 0.0142395 | 0.0093792 | 4.00E-07 | 0.73079 | 1229.59 | Hospitalized covid |
| rs10790976 | 11 | 128778315 | T | C | 0.111758 | 0.118333 | -0.0061579 | 0.0226516 | 0.013143 | 1.50E-07 | 0.63941 | 1290.52 | Hospitalized covid |
| rs117830939 | 8 | 88806713 | T | C | 0.047662 | 0.188806 | 0.017615 | 0.0337571 | 0.026484 | 8.50E-09 | 0.50597 | 1502.923 | Hospitalized covid |
| rs12203592 | 6 | 396321 | T | C | 0.219623 | 0.0470769 | 0.066996 | 0.0168804 | 0.017034 | 3.90E-09 | 8.38E-05 | 351.9347 | Hospitalized covid |
| rs12639198 | 3 | 187891885 | A | G | 0.284192 | 0.0758272 | -0.0055027 | 0.0160451 | 0.011292 | 4.40E-07 | 0.62604 | 1085.45 | Hospitalized covid |
| rs3130320 | 6 | 32223258 | C | T | 0.623606 | -0.0771445 | 0.0010095 | 0.0145878 | 0.011431 | 8.30E-10 | 0.92963 | 1296.916 | Hospitalized covid |
| rs34613444 | 2 | 58499373 | G | T | 0.058915 | -0.149106 | 0.025678 | 0.0303123 | 0.017695 | 3.50E-07 | 0.14673 | 1144.066 | Hospitalized covid |
| rs722266 | 11 | 92034702 | C | T | 0.817568 | -0.100221 | 0.032562 | 0.0184403 | 0.012099 | 7.80E-08 | 0.0071178 | 1391.168 | Hospitalized covid |
| rs748154 | 8 | 134708408 | A | G | 0.317744 | 0.0754683 | 0.0025251 | 0.015242 | 0.0098627 | 3.00E-07 | 0.79793 | 1145.939 | Hospitalized covid |
| rs78081797 | 3 | 94777466 | T | C | 0.060878 | 0.14461 | 0.053404 | 0.0319317 | 0.026084 | 4.20E-07 | 0.040621 | 1109.565 | Hospitalized covid |
| rs9287883 | 2 | 168396255 | A | G | 0.311151 | -0.0711087 | -0.0099901 | 0.0153409 | 0.010173 | 3.40E-07 | 0.32608 | 1005.581 | Hospitalized covid |
| rs10773497 | 12 | 128644754 | C | T | 0.478062 | 0.0728594 | 0.0010841 | 0.0142395 | 0.0044181 | 4.00E-07 | 0.80618 | 1229.59 | Covid |
| rs10790976 | 11 | 128778315 | T | C | 0.111758 | 0.118333 | -0.0060535 | 0.0226516 | 0.006677 | 1.50E-07 | 0.36461 | 1290.52 | Covid |
| rs117830939 | 8 | 88806713 | T | C | 0.047662 | 0.188806 | 0.013973 | 0.0337571 | 0.012544 | 8.50E-09 | 0.2653 | 1502.923 | Covid |
| rs12203592 | 6 | 396321 | T | C | 0.219623 | 0.0470769 | 0.010007 | 0.0168804 | 0.0073708 | 3.90E-09 | 0.17458 | 351.9347 | Covid |
| rs12639198 | 3 | 187891885 | A | G | 0.284192 | 0.0758272 | -0.0023315 | 0.0160451 | 0.0052016 | 4.40E-07 | 0.65399 | 1085.45 | Covid |
| rs3130320 | 6 | 32223258 | C | T | 0.623606 | -0.0771445 | -0.0067972 | 0.0145878 | 0.0048617 | 8.30E-10 | 0.16207 | 1296.916 | Covid |
| rs34613444 | 2 | 58499373 | G | T | 0.058915 | -0.149106 | -0.0040827 | 0.0303123 | 0.0083438 | 3.50E-07 | 0.62462 | 1144.066 | Covid |
| rs722266 | 11 | 92034702 | C | T | 0.817568 | -0.100221 | 0.010006 | 0.0184403 | 0.0057135 | 7.80E-08 | 0.07989 | 1391.168 | Covid |
| rs748154 | 8 | 134708408 | A | G | 0.317744 | 0.0754683 | 0.0042495 | 0.015242 | 0.0047551 | 3.00E-07 | 0.3715 | 1145.939 | Covid |
| rs78081797 | 3 | 94777466 | T | C | 0.060878 | 0.14461 | 0.001592 | 0.0319317 | 0.012251 | 4.20E-07 | 0.8966 | 1109.565 | Covid |
| rs9287883 | 2 | 168396255 | A | G | 0.311151 | -0.0711087 | 0.0064279 | 0.0153409 | 0.0047697 | 3.40E-07 | 0.17777 | 1005.581 | Covid |

**Supplementary Figure S1 Leave-one-out results of each COVID-19 trait on sepsis**

| 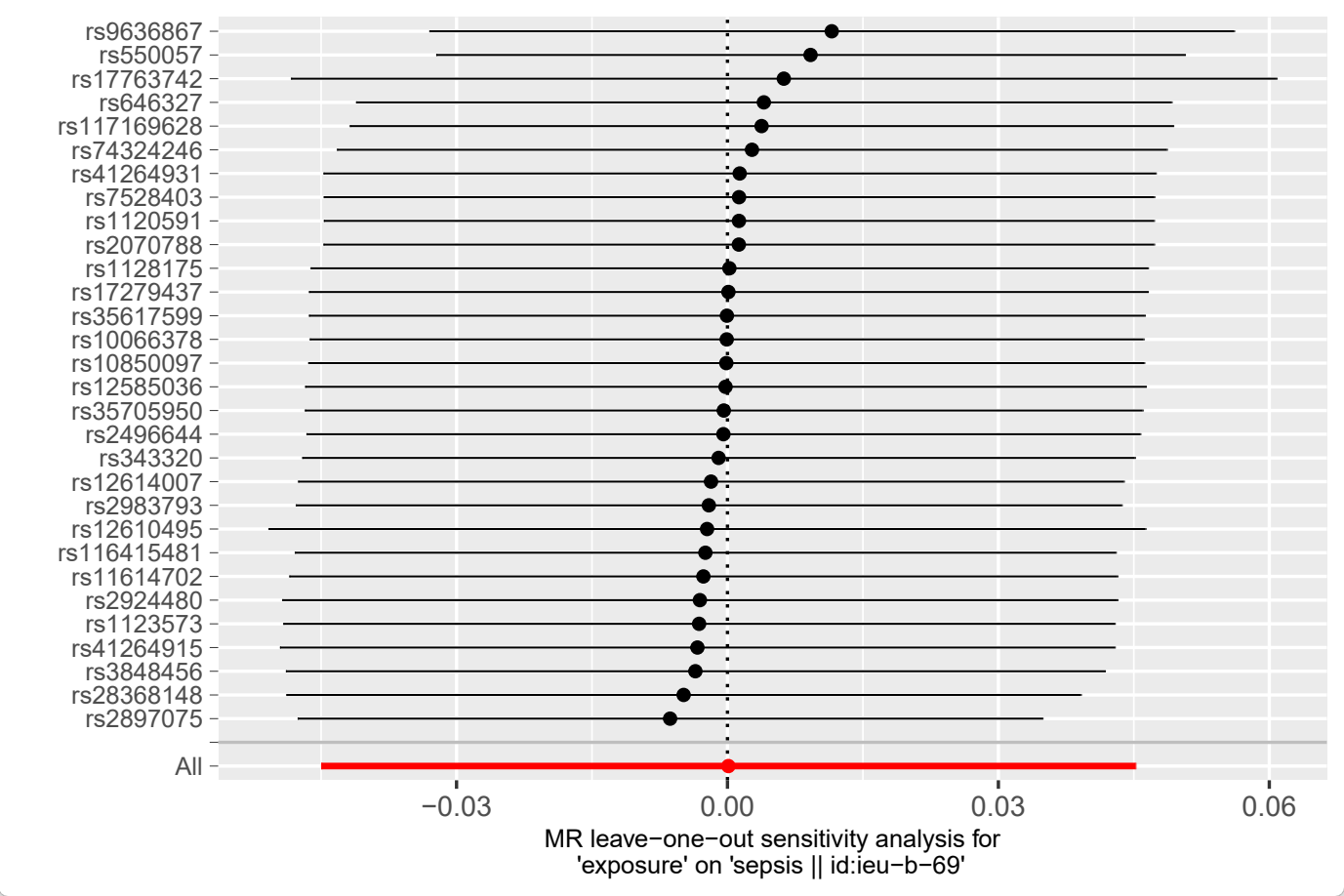 | 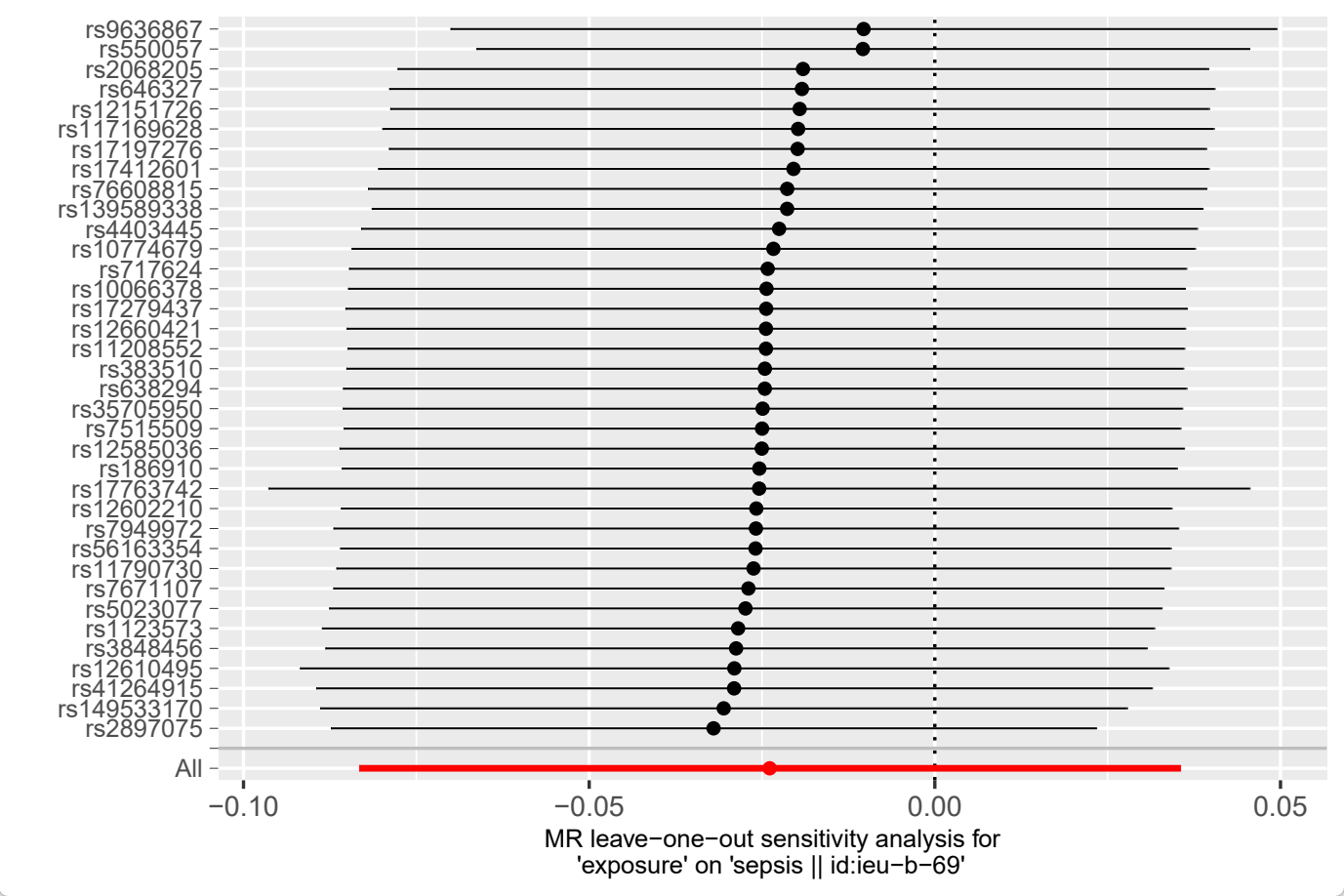 | 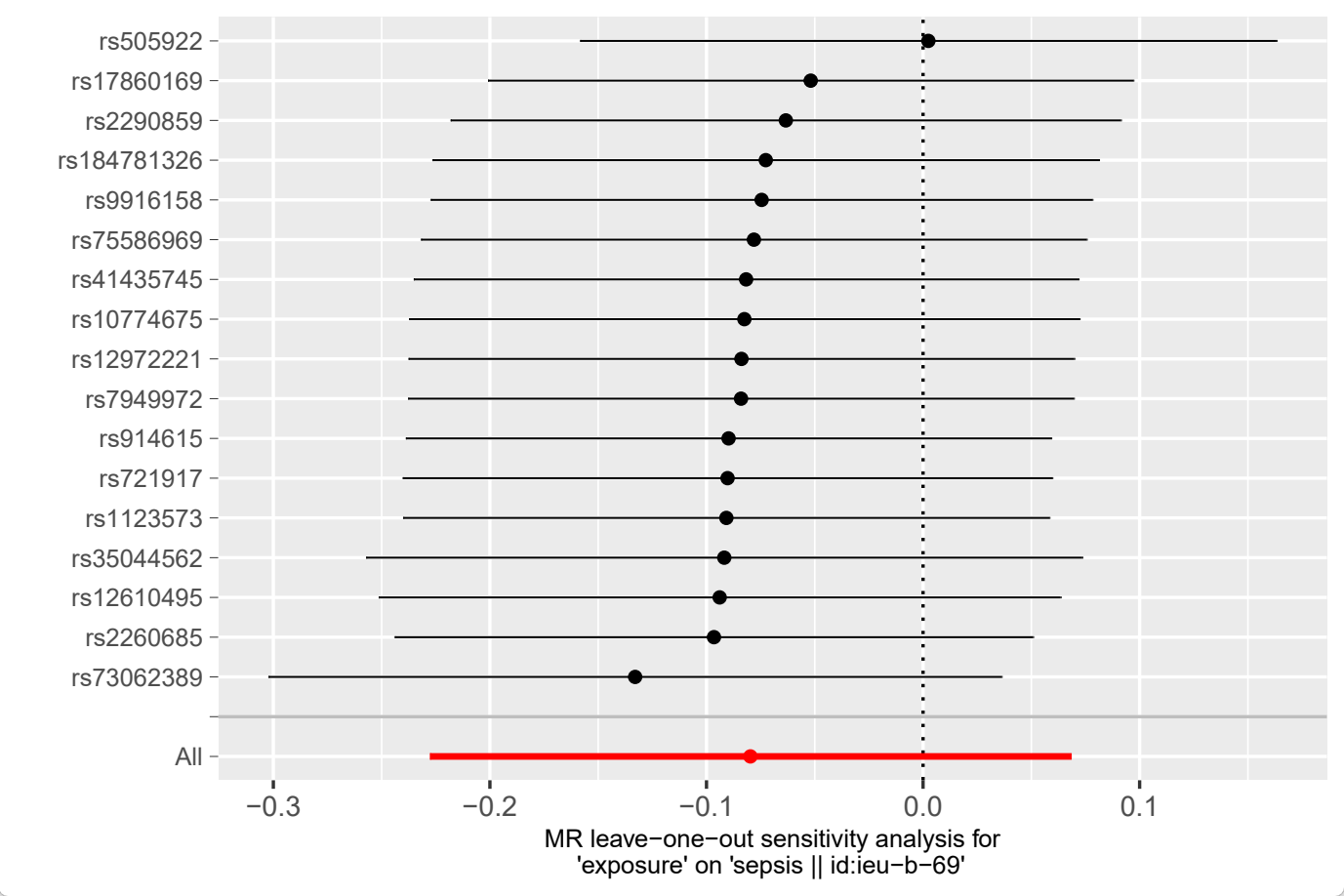 |
| --- | --- | --- |
| Leave-one-out analysis of very severe respiratory confirmed covid on sepsis | Leave-one-out analysis of hospitalized covid on sepsis | Leave-one-out analysis of covid on sepsis |

**Supplementary Figure S2 Leave-one-out results of sepsis on each COVID-19 trait**

| 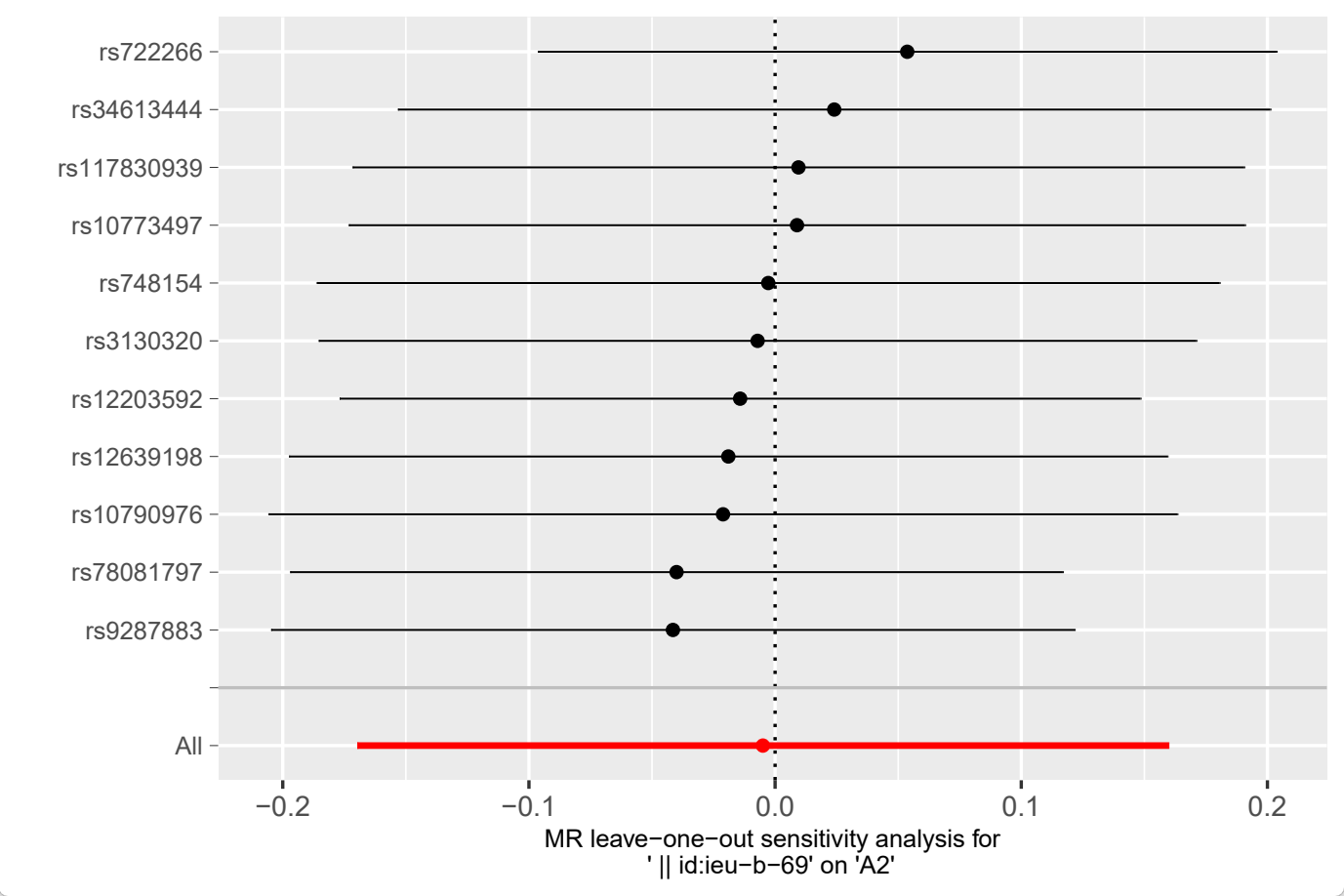 | 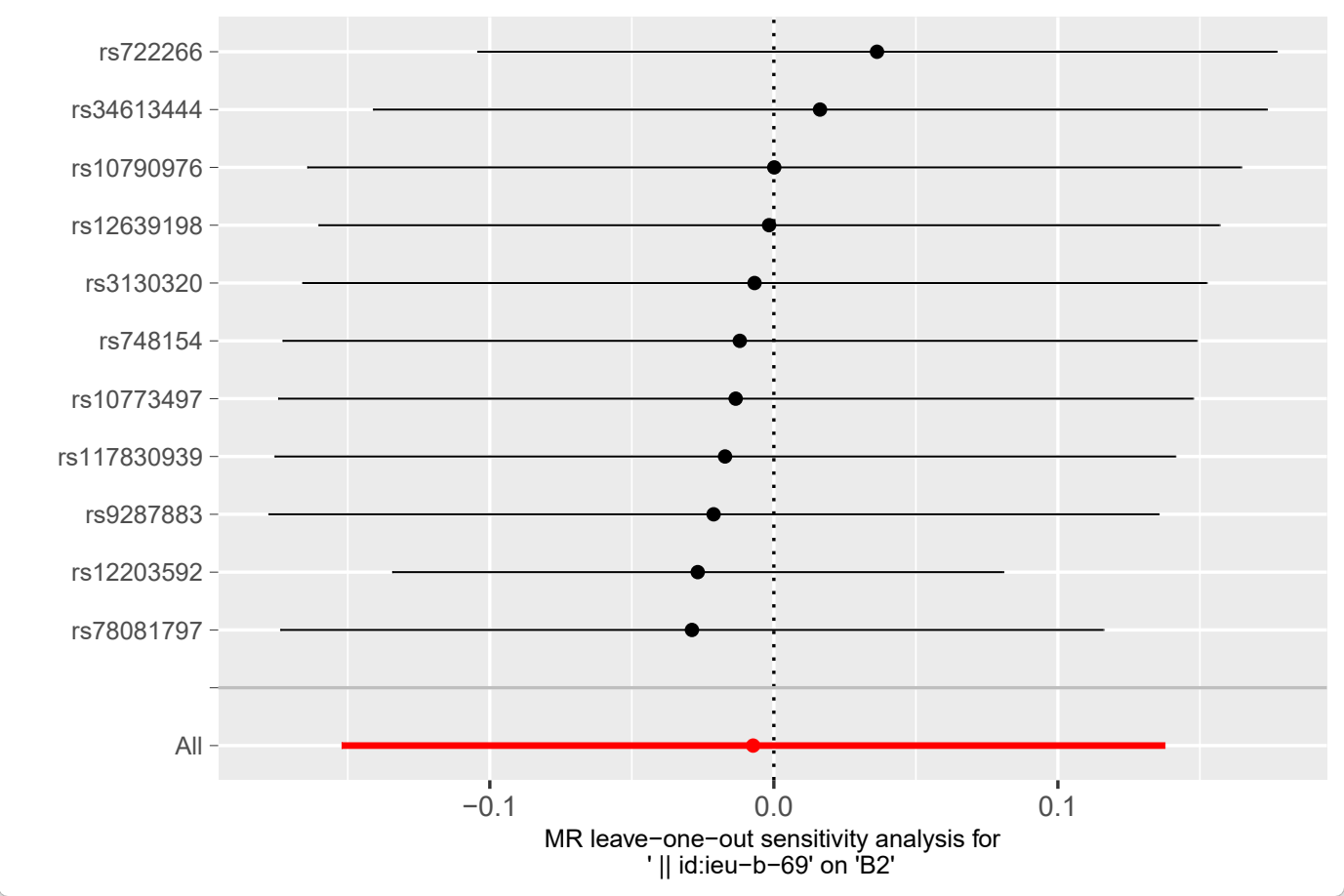 | 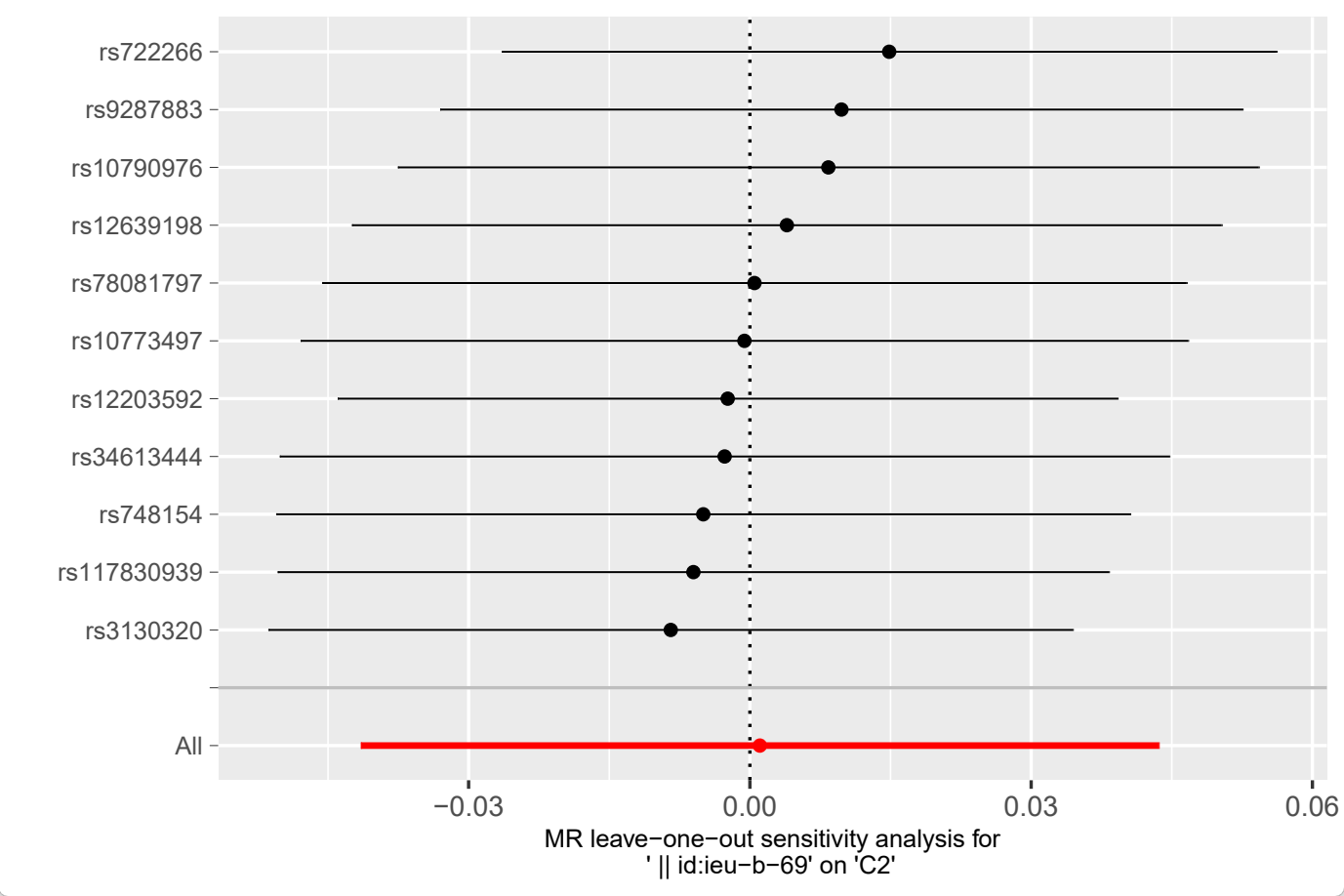 |
| --- | --- | --- |
| Leave-one-out analysis of sepsis on very severe respiratory confirmed covid | Leave-one-out analysis of sepsis on hospitalized covid | Leave-one-out analysis of sepsis on covid |
